# Exploring the construct of anticipatory stress in finding a job after residency training through cognitive interviewing: Implications for learner well-being and health workforce planning

**DOI:** 10.12688/mep.19559.1

**Published:** 2023-05-03

**Authors:** Sana Jawad, Megan Thomas, Kent Hecker, Aliya Kassam

**Affiliations:** 1 Undergraduate Medical Education, Cumming School of Medicine, University of Calgary, Calgary, Alberta, T2N 4N1, Canada; 2 Faculty of Pharmaceutical Sciences, The University of British Columbia, Vancouver, British Columbia, BC V6T 1Z3, Canada; 3 Department of Veterinary Clinical and Diagnostic Sciences, Cumming School of Medicine, University of Calgary, Calgary, Alberta, T2N 4N1, Canada; 4 Community Health Sciences Cumming School of Medicine, Health Sciences Centre Foothills campus, 3330 Hospital Drive NW, University of Calgary, Calgary, Alberta, T2N 4N1, Canada

**Keywords:** postgraduate medical education, stress, workforce planning, learner wellbeing

## Abstract

**Background:** Anticipatory stress (AS) is denoted by concern about future events for which there is little control. Most AS research has been physiological studies such as measuring salivary cortisol levels. Medical learners may experience AS regarding employment after residency, however AS a psychological construct across career stages has not previously been studied. The objective of this study is to explore the psychological construct of employment AS in medical students, residents, and former Program Directors (PDs).

**Methods:** Participants were recruited from a large Canadian medical school via purposive sampling. Semi-structured interviews with n=21 participants (six medical students, nine residents, and six PDs) were transcribed verbatim, and coded by two independent reviewers using thematic analysis.

**Results:** Participants agreed that financial, family, and geographical factors exacerbate AS, and it is mitigated by flexibility, social support, and being proactive. External support, job market saturation, and differences between medical specialities also influence AS. Perspectives unique to participant groups included: medical students reflecting on a hidden curriculum and preoccupation with proximal issues over distal concerns of employment; residents experiencing competing residency program demands; former PDs finding that resident competency, yearly hiring fluctuations, and existing stress impact AS. Consequences of AS include physical and psychological manifestations, performance anxiety, and pursuing additional training.

**Conclusions:** Perceptions of AS vary by medical career stage. Individual, program and systems-level changes can help manage and address the underlying cause of AS: an unreliable job market for physicians. Correcting the mismatch between residency positions and job openings may be a proactive, preventative approach.

## Introduction

Choosing a specialty and securing a job after residency can be a source of stress for medical students, resident physicians (residents) and fellows. A significant factor associated with specialty choice in medical school is positive experience as an undergraduate medical learner. Medical learners who choose a different specialty than those they preferred in medical school are influenced by domestic circumstances and working hours ^1– 3^.

A competitive job market for physicians, especially in resource-intensive specialities such as radiation oncology and surgical disciplines, is prevalent in existing literature ^4– 9^. Employability concerns were the second most common reason for Canadian general surgery residents to consider leaving their residency training (following poor work-life balance) ^5^. Among newly graduated pediatric cardiologists, all respondents in a 2019 study reported difficulty finding work; nearly 40% reported it was more difficult than they expected, and 63% of respondents said it was the most stressful issue during their residency ^6^. The Employment Study released by the Royal College of Physicians and Surgeons of Canada (2013, follow up report in 2019) contextualizes and describes these trends ^7, 8^. Specifically, from 2011–2019, new specialists reported that the unemployment rate was between 11–18%. This is substantially larger than the unemployment rate for all Canadians (7%) ^9^.

Anticipatory stress (AS) is denoted by concern about future events for which there is little control. It is a form of perseverative cognition that may manifest as undue worry or rumination and is associated with negative outcomes through oxidative damage and hypercoagulable states yet most research to date has examined physiological correlates as opposed to exploring the psychological construct of AS ^10– 14^. AS has also been shown to impair decision making abilities and lengthen decision-making time, both of which are relevant for the daily tasks of a physician ^14^. Due to the potential negative impacts of AS on medical learner well-being and performance, describing this construct could assist in mitigating impacts of AS on medical learner well-being. For example, AS of potential unemployment may be a large factor that motivates newly graduated physicians to pursue subspecialized training. In 2017, 43% of new specialists completed sub-speciality or fellowship training, and over half of these individuals pursued additional training because they believed it would make them more employable ^15^.

Our study explored the psychological construct of AS in medical students, residents and former program directors regarding securing employment following postgraduate training from the perspective of being a resident. We hypothesized that 1) AS is modulated by other personal and professional factors, such as one’s financial circumstances and medical speciality of choice, that influence individual stress levels and 2) Each of the three groups would have different perspectives on AS related to finding employment in their speciality of training, with residents having the greatest AS because they are in closest proximity to completing their training.

## Methods

### Study design

We used pragmatism as the theoretical construct for our research study, and the underlying premise of this theory is that knowledge is created from socially shared experiences and is influenced by our beliefs, habits, and interactions with others ^16^. In our study, we first identified a common problem among medical learners: job prospects after residency training and sought to explore how it could impact learner well-being. We were also interested in how it could be measured for future studies. Cognitive interviewing is a method for empirically studying how participants mentally process and respond to survey questionnaires by thinking and talking aloud ^17^. This helps delineate how participants understand the construct being measured. To explore the construct of anticipatory stress, we applied the cognitive interviewing technique to the Perceived Stress Scale (PSP) ^18^ and the Primary Appraisal/Secondary Appraisal (PASA) ^19^ scale, the latter of which was adapted for use in the context of finding a job after residency training (see *Extended data*) ^20^. For example, the PASA scale is based on the transactional stress theory ^21^ and divides the assessment of participants' primary appraisal of stress (whether the stressor is in fact a threat to the participant) and participants secondary appraisal (whether the participant can cope with the stress) regarding a specific challenging potentially stress-inducing scenario ^22^ which in our study was delineated by finding a job after residency training. As such, questions were adapted to include the specific scenario related to finding a job after residency.

### Participants

Participants were recruited from Undergraduate Medical Education (UME) and residency programs at University of Calgary’s Cumming School of Medicine via purposive sampling by sending emails through listservs. Individual semi-structured interviews with n=21 participants (six medical students, nine residents, and six PDs) were conducted from June 2016 – March 2017 by A.K in an office with no other members present. 

### Data collection

A semi-structured interview guide was also used to explore how participants define AS, understand factors leading to stress associated with residents finding a job after residency, and to explore the manifestations of AS experienced by participants. This was developed and reviewed by the study collaborators which included a resident physician, two staff physicians as well as two medical education researchers (non-clinical academic staff with doctorates in medical education (K.H.) and health services research (A.K.). Participants did not have a prior relationship with the interviewer, were aware of why the study was being conducted and A.K.’s role in the study. A full list of our interview questions can be found under *Extended* data ^20^. Interviews were audio-recorded and transcribed verbatim and participants received a twenty-five-dollar gift card as a token of appreciation. Interviews lasted between 45–60 minutes in duration and coded by two independent reviewers (S.J. and M.T.) using thematic analysis in 2019 and 2021. We quantified the frequencies of different themes. A.K. reviewed all transcripts and K.H read a sub-set of transcripts to determine data saturation. Discrepancies in coding were discussed with A.K. until consensus was achieved.

### Data analysis

Thematic analysis is a widely used analytic method for qualitative research and allows us to code patterns across interviews. We followed the six key phases of thematic analysis outlined by Braun and Clarke ^23^ using Microsoft Excel 1) Familiarization with the data by transcribing all of the interviews verbatim, 2) Generating initial codes by systematically reviewing the interviews and coding interesting features of the data, 3) Searching for themes across the codes, 4) Reviewing and refining the themes, 5) Defining and naming the themes, and 6) Reporting the themes by presenting them in a table format with a corresponding written analysis. A condensed codebook is presented under *Extended data* ^20^. Participants were not asked to provide feedback on the data however reflexivity was maintained by having two independent coders, and the principal investigator (A.K.) being cognizant of her bias as the research lead in the Office of Postgraduate Medical Education and learner advocate in the collection of data and presentation of the results in the manuscript.

### Ethical considerations

The Conjoint Health Research Ethics Board (CHREB) approved this research (REB14-1267/REB15-2816) and informed consent was obtained from all participants by reviewing the study, seeking permission to audio-record the interview and signing the consent form. Participants were told that the data would be de-identified, analyzed in aggregate and presented in the format of themes supported by quotations.

## Results

A total of N=21 interviews were conducted with medical students, resident physicians, and former program directors. From the n=6 medical students, 50% identified as women and 50% identified as men, 50% fell in the 25–29 years old category (two were aged 20–24 and one was 30–34), and they were dispersed equally through all years in a three-year undergraduate medical education program. In our sample of n=9 resident physicians, 62.5% identified as women while 38.5% identified as men, 75% were aged 30–34 years old (one participant was 25–29 and two were 35–39), and they ranged in level of training from PGY1 (12.5%), PGY2 (37.5%), PGY3 (12.5%), PGY4 (12.5%) and PGY5+ (25%). The residents came from diverse specialties including surgery, radiology, pediatrics, family medicine and internal medicine. From the n=6 former Program Directors interviewed, 60% identified as women and 40% identified as men, and they served as former Program Directors for surgical, pediatric and internal medicine specialties. Thematic results are presented below supported by medical student (MS), resident (R), and program director (PD) quotations.

### Differentiating anticipatory stress from other forms of stress

When asked to define AS, participants remarked that it is stress related to future events and is associated with a lack of control. In the words of one participant, “It’s not necessarily an acute active stress right now, but it’s the stress created thinking about or leading up to an event” (MS06). This differentiates it from other forms of stress, which are related to more imminent or tangible concerns. Although the temporal relationship to the stressor differs, many of the physical and psychological manifestations are the same. This includes insomnia, anxiety, and somatization into symptoms such as headaches and back pain. 

### Anticipatory stress themes unique to medical students

The themes we identified fell into three categories: 1) factors that worsen AS, 2) factors that mitigate AS, and 3) consequences of AS. Common themes were that the level of AS experienced is influenced by personal factors such as financial responsibilities, familial commitments and flexibility to relocate, along with institutional and systems-level factors such as availability of support, job market saturation, and intrinsic differences between medical specialities. Alongside these common themes, there were some notable perspectives unique to the participant stage of training ( Table 1).

**Table 1.** Themes unique to medical students (n=6), residents (n=9) and former Program Directors (n=6), elicited during cognitive interviewing and semi-structured interviews about Anticipatory Stress (AS).

Theme		Illustrative Quotations
Medical Student	Hidden Curriculum	*There are times in our education where we’re pushed to go in a certain direction and we aren’t * *able to fully explore, because of how the program is structured or what is emphasized in* * course material*. (MS02)
	Limited Options	*I feel like I don’t have options*. (MS02)
	Residency Matching Stress	*I don’t think there’s any medical student in their final year training who is going through the* * CaRMS [Canadian Residency Matching Service] process who isn’t experiencing the stressors of* * the match*. (MS06) *A match is designed to match medical students to residency placements. And that marriage in* * a sense is critical to getting a job after residency… whatever residency you’re in directly links to* * what your career prospects are going to be*. (MS06)
Resident	Multilayered Stress	*There’s the stress of what you don’t know and the sort of imagining stress of “okay, will I be able* * to find a job?” …As you start to look for jobs, there’s additional layers of stress involved with * *“okay, now I tried to get a job somewhere and they don’t have any capacity.” “…I couldn’t get a* * job there, so I’m going to start to look at other places….” There’s actually I find different layers* * to it, depending on which stage you’re at. But it’s all stressful; it’s an incredibly stressful time*. (R01)
	Post-employment Stress	*I actually have a job already, so there’s stress kind of getting that sorted out, but now that I * *have the job… making all the arrangements that are necessary to go there… there’s so many* * balls in the air right now and it is a very uncertain period, even though to some degree I have* * some certainty. I have a job and I know there will be work for me, I feel like it’s still very stressful* * somehow.* (R01)
	Competing Academic Demands	*There’s other demands too. Like right now we have a big research project… it’s additionally* * stressful in that you have all these emotional and psychological things going on, but you* * just have no time to deal with any of it*. (R01) *Another big stress is your Royal College exams. You’re studying the hardest thing you’ve ever* * studied, but at the same time trying to find a job… You’re trying to find a job, but you’re always* * thinking, “well, this is conditional that I pass my exam.”* (R06)
Former Program Director	Resident Preparation	*Some residents you feel have done everything and they really deserve to get hired and then* * there’s some residents that honestly… they just don’t try very hard. And it is seen… they just were* * regarded as being people that kind of did the bare minimum to get by and there’s a reason* * why nobody is gunning to hire them.* (PD01) *What struck me… was how ill-prepared the applicants were…They often knew nothing about the * *program here or the city I just thought it was really unfortunate that people were so---these are* * thirty-five-year-old people, right? That they were so poorly prepared and it was actually in some* * ways a reflection of the way we educate people. That we don’t say to them you know, “this is a* * job interview. There’s eighty applicants, there’s four spots in my center… it’s very competitive….”* * I think the reality is that once you get into medical school, a lot of it is kind of automatic. You* * apply for stuff and its writing application forms, the interviews aren’t---but by the time you get* * to fellowship or job interview you have to have those skills. And most of them don’t.* (PD02)
	Hiring Fluctuation	*It’s not always clear how many people are going to get hired, and sometimes they do a hiring* * cycle and actually don’t hire anybody. So that can be disappointing to people because they * *actually go through interviews and then nobody is hired. There’s quite a bit of fluctuation.* (PD01)
	Program Director Personal Opinions	*It’s only natural that I’m on staff, so I’m going to have my personal feelings about how I would* * rank them* (residents) *… It is stressful because sometimes you kind of know… that a certain* * person isn’t going to get hired, just based on the scuttlebutt of your colleagues and friends* * around you*. (PD01)
	Trickle-down stress	*Residents who are a year away from graduating, if the cohort ahead of them didn’t fare well, * *and there wasn’t a lot of jobs that year, then there’s a lot of trickle down stress that occurs….* * just because the cohort ahead of you didn’t get hired, doesn’t necessarily mean that there* * aren’t jobs.* (PD01)

Medical students reported a hidden curriculum and feeling that their UME course structure influenced their future career decisions. They also reported feeling a lack of options, and multiple students were more concerned about proximal concerns, such as the upcoming residency matching process, rather than the distal concern of job prospects. Students reflected on how concern for matching to residency, through the Canadian Residency Matching Service (CaRMS) ^17^, is inextricably related to anticipatory stress of employment, as “whatever residency you’re in directly links to what your career prospects are going to be” (MS06).

### Anticipatory stress themes unique to residents

Two resident-specific themes that emerged were: reflecting on the multilayered nature of anticipatory stress and how each stage in their medical career brings a different form of stress related to the next step of their professional journey ( Table 1). Balancing the stress of job searching alongside various other academic commitments, such as research and completing Royal College exams at the end of their residency training, led residents to experience multiple layers of AS. As one resident aptly summarized, “You’re trying to find a job, but you’re always thinking, ‘well, this is conditional that I pass my exam’” (R06). Residents also felt that their AS extended beyond the worry of *securing* employment, but also into stress related to feasibility, administrative tasks and possible relocation for job offers. One resident who had already secured a job commented, “there’s so many balls in the air right now and it is a very uncertain period, even though, to some degree I have some certainty. I have a job and I know there will be work for me, yet I feel like it’s still very stressful somehow” (R01).

### Anticipatory stress themes unique to former program directors (PDs)

Discussion points specific to the former-PD category included resident incompetency and reflecting on systems-level causes of AS ( Table 1). Former PDs commented on some residents being ill prepared for employment after residency, either due to lack of effort throughout the course of their training, or poor interview skills and preparation when being interviewed for jobs. Former PDs also remarked that the hiring process can be ambiguous and unpredictable; job positions may be created for outstanding applicants even when there did not appear to be an initial need, or medical institutions may interview applicants and ultimately not hire anyone. Former PDs had the perspective of overseeing multiple cohorts of residents and recognized that poor employment prospects for one cohort of graduating residents causes a wave of anticipatory stress in future cohorts, however this is an unfounded fear as the hiring cycles vary substantially each year ( Table 1).

### Common theme: Factors that contribute to anticipatory stress

While we have highlighted the unique aspects of AS identified by medical students, residents, and former PDs, most themes overlapped across the three groups ( Table 2).

**Table 2.** Common themes about contributing and mitigating factors, and consequences of Anticipatory Stress (AS), arising during cognitive interviewing and semi-structured interviews across three career stages: medical students (n=6), residents (n=9) and former Program Directors (n=6).

Theme	Illustrative Quotations
Contributing Factors	
**Speciality ** **Dependent**	*There’s a perceived lack of jobs. I don’t know if that’s real or not and it depends on the specialty. For example, * *in orthopedics there are jobs in community settings. Those residents grow up through an academic setting * *and I get the sense that their lens for a job is an academic setting and not necessary community setting. So * *they’re not willing to move or consider those jobs*. (PD03)
**Psychological** *Uncertainty Lack of * *control*	*Lack of jobs today does not equal lack of jobs in seven years. And likewise, jobs that are available today does * *not mean that they’re going to be there… however long your residency is. You have no way of knowing*. (MS01) *It’s just kind of that unknown; that element of the unknown and no one really plans and maybe it will work* * and maybe it won’t. You just don’t know*. (R05)
**Social** *Family Circumstances*	*One factor for stress will be wherever I go - can my husband also come, and can he get a job?* (MS01) *For me, having a child has definitely swayed my decision to not pursue a fellowship… this would also apply if * *you had a sick family member… changing your goals in residency in terms of pursuing something general or* * something rural or something in a different province because that’s where your family member is*. (R03)
**Perceived lack of** ** jobs**	*There’s chatter that occurs across the country and people are hearing “oh, our site isn’t hiring, our site isn’t* * hiring,” so people get anxious about that*. (PD01)
**Logistic** *Location preferences*	*The vast majority of the trainees that we train are keen to stay and for a lot of those reasons. This is where* * their lives are and they’re adults now…They’re in their thirties and they’re married with children and uprooting * *them and trying to find a place to be is incredibly stressful for them*. (PD01)
**Financial** *Debt and * *responsibilities*	*Older students like myself… gave up their fulltime jobs to come back to school and that is a very big financial * *stressor. Or have just been in school forever… and have student debt from having three previous degrees.* (MS01) *Knowledge that we’re coming in with the highest debt loads and we’re probably going to have the lowest * *earning by the time I’m done my career*. (R08)
**Inadequate ** **supports** *Lack of information* *Lack of institutional * *support*	*It seems like you automatically just don’t have a job once you finish residency and medical school. And that’s* * not often related to us, so there’s difficulty in navigating that all by ourselves.* (MS04) *It’s just hard to know what the right decision is, given that I feel like I don’t truly understand the ramifications * *of some of the decisions that I make*. (R01) *It seems like not enough is being done by the people that would have to take action to help fix the job market* * problem. So in orthopedic surgery, at least in Halifax, are taking just as many residents as they always did * *every year and no one is getting a job. Nothing is being done to correct the actions.* (R04) *I don’t think we as program directors have a lot of national support… And I don’t think that’s specific to * *PGME… In my opinion, they haven’t done a really good job of maximizing opportunities to help the program* * directors find positions or advertise positions for residents. And that had been brought up at the [Royal * *College of Physicians and Surgeons of Canada], but the college’s position is “that’s not our mandate; our * *mandate isn’t finding jobs, our mandate is training residents,” and I can see that. But then there should be * *somebody’s mandate to help on a national level and recognize that there are lots of trainees coming out who * *need work*. (PD05)
**Systemic** *Changing Landscapes* *Oversaturation* *Competition*	*There was a time when universities would say to you, ‘you will have a job here when you come back.’ Nobody* * would say that now. So now the best that they can hear is you know, ‘We’re very interested. We cannot make* * you any promises; keep in touch….’ Those are people who, twenty years ago, would have been given a* * contract*. (PD02) *Fifteen years ago… you really just pursued what you wanted to do and what you liked. And I think that’s* * becoming not a secondary factor, but it’s almost on par with what the job map is out there for people.* (PD04) *Something I’ve noticed and not just among me, but also among my classmates, is that once we hear that the* * job market is super-saturated and we might be doing a longer residency than we intended and fellowships. * * We automatically disregard those programs.* (MS04)
Mitigating Factors	
**Social** *In-group ties* *Support systems*	*Most physicians can get jobs by essentially word of mouth*. (R09) *Sometimes there’s just fellows that have trained, who people really, really like and think are awesome people * *and even if you’re not looking for somebody actively, they kind of make room for them.* (PD01) *It’s not like applying for a job at IBM, where if there’s a job there’s a job. What often happens in academic* * medicine is somebody really excellent finishes the program and even though nobody is retiring and there’s no * *real need to recruit another person… they make room. Now, is that fair? I don’t know, but that’s actually how* * it works*. (PD02)
**Proactive Choices**	*We need a plan. What fellowship are you applying to? You have to have contacts. You have to start thinking* * about do you want an academic career? Do you not want an academic career? Where are you going to go? * *Are you prepared to leave Canada? Like I used to have like a whole list. So by the middle of your second year* (of residency) *you need a plan and you need to start making contacts.* (PD02)
**Flexibility**	*I don’t have any kids and no one really relies on me, so I can go around and do locums. I’m very confident I’ll * *be able to support myself…. I would strongly prefer to have a job at the end of residency, but if I don’t it’s not * *going to be the end of the world*. (R04)
**Passion vs. ** **Availability**	*Some people want - and I’ll use orthopedic surgery as an example - …really want that as a career, but they * *recognize that there’s very poor job prospects at the end, so it's then that disconnect between what I want to* * do and what I'm passionate about, but will it actually get me a job?* (MS06) *Some interests don’t lend themselves to find work that easily. So then you worry, “oh, should I pursue what I * *like or what’s going to get me a job?”* (R05)
Consequences	
**Manifestations** *Physical* *Psychological*	*In my experience of stress or anticipatory stress I feel a little bit sick to my stomach. I might have changes in* * how my body feels*. (MS01) *It might be a racing heart, it might be chest pains; it might be you’re diaphoretic or sweating, it might be just * *that angst nervousness and restlessness*. (MS06) *Anxiety and just like lack of motivation can lead to the onset a lot of depressive symptoms as well and feelings* * that you aren’t getting what you expected or you don’t fully have control over what you thought you did*. (MS02)
**Pursuing ** **Additional Training**	*I interact with the residents in cardiac surgery and there was a period of about five or six years when it was* * simply understood across Canada that there were zero jobs, zero. And so people were doing things like * *Masters Degrees and PhDs mostly as a way of treading water until something changed*. (PD02)
**Performance** ** Anxiety**	*A lot of times they feel like for the last couple of years of their training, like every single day is a job interview. * *Like they sort of feel like that stress is there all the time*. (PD01)
**Insomnia**	*You sleep less, you’d be a little more anxious*. (MS04)
**Variable** ** Presentations**	*It depends on the person...everyone is going to react differently in terms of how they handle anticipatory* * stress or stress in general*. (MS01)

 Participants recognized that the level of AS experienced by a medical learner depends on their speciality of interest, as some specialties inherently have more competitive job markets than others. Psychologically, elements that contributed to an individual’s AS were fear of the unknown, and a lack of control over the situation. Personal factors, such as having a family, a strong desire to secure employment in a particular location, and increased financial responsibilities and debt, were mentioned with the greatest frequency as contributors to AS.

 A perceived lack of jobs also contributes to heightened AS, and all three groups felt inadequately supported to meet the needs of AS. Medical students and residents felt that they were provided with insufficient information and guidance regarding career planning, while former PDs felt an absence of support from national key stakeholder organizations such as colleges and regulating bodies. One former PD remarked, “that had been brought up at the [Royal College of Physicians and Surgeons of Canada], but the College’s position is ‘that’s not our mandate; our mandate isn’t finding jobs, our mandate is training residents,’ and I can see that. But then it should be somebody’s mandate to help on a national level and recognize that there are lots of trainees coming out who need work” (PD05).

Similar to this theme, participants commented on systemic factors that contribute to AS, such as increased competition in the medical field and oversaturation of certain specialties. Former PDs acknowledged that due to changing landscapes of the physician job market; securing employment has become more challenging and uncertain than in the past. One former PD candidly recalls, “There was a time when universities would say to you, ‘you will have a job here when you come back.’ Nobody would say that now. So now the best that they can hear is you know, ‘We’re very interested. We cannot make you any promises; keep in touch….’ Those are people who, twenty years ago, would have been given a contract” (PD02).

### Common theme: Factors that decrease anticipatory stress

 In contrast to a strong preference for residing in a certain location, which contributes to AS, flexibility was reported as a protector against AS ( Table 2). Participants who were open to relocating or working as a locum for a few years before securing full-time employment reported experiencing less AS about job prospects. Two main types of social support were also protective: 1) In-group ties to other medical professionals, as this helps new trainees secure employment through positive interactions with future employers and word-of-mouth connections, and 2) personal social support networks, such as a supportive partner, family, and friends.

 Proactive choices were also found to be protective against AS. This involves anticipating the career planning process and social connections that a trainee must make in order to secure their preferred type of employment and taking initiative well in advance.

 Another theme related to mitigating AS was Passion vs. Availability. While some participants stated that job market availability greatly influenced their career decisions, most felt that their passion towards a particular speciality in medicine surpassed the fears of unemployment. For this reason, most residents and former PDs felt content with their career decisions despite the AS they experienced.

### Common theme: Consequences of anticipatory stress

 Participants reported both physical and psychological manifestations of AS, along with insomnia, performance anxiety, and a desire to pursue additional training to be more employable ( Table 2). Participants report the following psychological manifestations: anxiety, insomnia, depressive symptoms, lack of motivation. Physical manifestations included: restlessness, chest pains, sweating, racing heart, nervousness, headaches, and fatigue ( Table 2). Participants tended to attribute the physical manifestations of AS to be the somatization of psychological manifestations.

Participants across all three groups also recognized that the manifestations of AS vary widely between individuals. Regarding performance anxiety and the constant pressure that residents face, one former PD remarked, “A lot of times they feel like for the last couple of years of their training, like every single day is a job interview. They sort of feel like that stress is there all the time” (PD01).

## Discussion

In this study, we explored perspectives of employment anticipatory stress after residency in medical students, residents, and former residency Program Directors (PDs). Our key findings include describing physical and psychological manifestations of AS such as insomnia, performance anxiety, and pursuing additional training, as well as identifying contributing and mitigating factors for AS. A perceived lack of jobs, inadequate institutional support, changing landscapes of the job market, and personal factors maintain and exacerbate AS. These personal factors include financial strain, family connections to an area, and unwillingness to relocate. In contrast, factors that decreased AS were flexibility, being proactive with career planning, social connections, and passion for a speciality. There was consensus around AS changing with respect to where on the continuum of learning and practice participants were situated.

These findings are in line with other studies, which examine resiliency and stress management in assumed healthy participants such as medical learners ^24– 31^. Residents in particular face high levels of stress, which comes in the form of different stressors. Commonly reported stressors in residency include fatigue, financial strain, family circumstances, and a lack of time; these factors overlap with exacerbators of AS that we have identified. Stress contributes to increased likelihood of burnout (a syndrome consisting of emotional exhaustion, cynicism, and inefficiency in response to chronic emotional and interpersonal work-related stressors ^26^) among residents, which worsens their responses to stress ^26^.

A targeted approach based on the cognitive pathways involved in AS may be useful in medical education. As a form of perseverative cognition, AS contributes to a prolonged cognitive representation of stressors and sustains the stress response ^25^. This cerebral aspect of the stress response is emphasized as a maladaptive thought loop that can be addressed with cognitive behavioural therapies ^24^. The interplay between cognitive and physiological processes is further emphasized by a recent study showing that participants with higher expectancies of being able to deal with a stressful situation showed a more positive anticipatory cognitive stress appraisal and lower cortisol response to stress ^25^. 

While the prior findings discuss AS in general contexts, our study takes the unique approach of examining AS in medical students, residents, and former PDs. Interviewing PDs provides a different lens by which to describe AS since they are involved in selecting medical students to join their respective residency programs, and in training those residents to enter a continually evolving job market. While PDs do not control the flux of this market, their role could encompass a duty to anticipate job market changes and prepare their residents accordingly, which is critical for resident well-being during training and ensures the successful transition of residents to independent practicing physicians.

Our data suggests that while PDs are consulted by residents for job advice, many feel unprepared in providing appropriate advice to assist. This indicates that either PDs require greater support or resources to meet residents’ concerns, or that this task needs to be delegated to another source, such as a career counselor or coach. PDs agree that there is overtraining of residents in certain medical specialties, but complex factors prevent them from decreasing their program sizes. As both learners and care providers, residents are admitted not only for their own training, but also to fulfil medical services and comprise a large part of the healthcare workforce ^28, 29^.

There are limitations to this study. Generalizability is limited as data were collected from a single site, and there was no long-term follow up. Future studies that sample multiple sites and incorporate a longitudinal design could build upon our findings and elucidate practical implications, such as whether experiencing AS influences future career decisions. Many participants report stress over inconveniencing their partners or family members due to their profession. From our findings, research into how to mitigate AS is a logical next step. Mitigating AS is important for improving the quality of life and well-being of medical learners but also for ensuring patient safety due to the detrimental impacts of AS on executive functioning and decision making.

 Future studies could incorporate a validated questionnaire to examine any potential confounders for experiencing AS, such as personality characteristics or mood disorders. Determining if an optimal level of AS is conducive for motivation related to speciality selection and future employment is also warranted, in other words, is all stress bad? Finally, in addition to the physiological markers described, brain activity could be measured and tracked as another biomarker for AS ^30, 31^.

Steps to address AS can take place across various levels. At the individual level, medical learners can ensure they are comfortable with professional networking, CV building, and job searching. At a program level, PDs could play an active role in advocating for residents and share their own experiences. At the systems level, healthcare systems as well as governing bodies such as the colleges could monitor resident employment trends systematically and regularly and use that data to forecast physician supply/demand. This information could be shared with residency programs. Postgraduate Medical Education (PGME) Offices could also develop curricula around these topics. While addressing AS may be important, it is worth noting that this does not address the underlying cause of the stress. Lessening resident anticipatory stress is a reactive approach, while correcting the mismatch between residency positions and job openings is a proactive, preventative approach for resident well-being and job security. This is particularly important given the healthcare provider shortage since the COVID-19 pandemic.

## Conclusion

This study examines the construct of anticipatory stress and finding employment after residency training through the perspectives of medical students, residents and previous PDs. Many trainees expressed anticipatory stress regarding finding a job, and some factors that influenced this were: lack of job market information, willingness to relocate, financial and family situation and the learning environment. Program directors shed light into systems-level causes that perpetuate a culture of AS; namely, a fluctuating and unpredictable job market, lack of national data collection and support to assist with matching trainees to employment, and insufficient resident preparation. Job market data surveillance and reporting, along with transparent career development initiatives to assist residents with securing employment, are warranted to ensure learner and health workforce needs are met.

## Data Availability

The data that support the findings of this study are available on request from the corresponding author, [AK]. The data are not publicly available due to information that could compromise the privacy of research participants and as such requests for the data must be approved by the institutional ethics board before access can be obtained. OSF: Exploring the construct of anticipatory stress of finding a job after residency training through cognitive interviewing: Implications for learner well-being and health workforce planning. https://doi.org/10.17605/OSF.IO/XQE2Z ^20^. This project contains the following extended data: Condensed codebook_Anticipatory Stress.docx Supplemental Material.docx Data are available under the terms of the Apache license 2.0.
