# Supplementary material for: Exploring the construct of anticipatory stress in finding a job after residency training through cognitive interviewing: Implications for learner well-being and health workforce planning
Source: MedEdPublish (2016). 2023 May 3;13:25. [Version 1] doi: 10.12688/mep.19559.1 (PMC10594048; doi:10.12688/mep.19559.1)
Supplement: Peer review history

# Reviewer report: Reviewer response for version 1

**DOI:** 10.21956/mep.20953.r34829

**Published:** 2023-10-23

**Authors:** Vera Meeusen

**Affiliations:** 1 The University of Queensland, Saint Lucia, Queensland, Australia

It would be good if the authors could explain psychological stress and its symptoms a little bit more in the introduction section. The sentence about cerebral aspects in the discussion section should be in the introduction section as it was not studied in this research project.

 Maybe the authors can address the causality of stress as well. This study does not explain what increases stress: is it the financial situation or family that increases AS for employment or other way around. It would be interesting the discuss this in the discussion section. It would also be interesting to benchmark these findings with findings in other sectors, outside the medical arena as this problem is recognised in many different areas..

Have any limitations of the research been acknowledged?

Yes

Is the work clearly and accurately presented and does it cite the current literature?

Yes

If applicable, is the statistical analysis and its interpretation appropriate?

Yes

Are all the source data underlying the results available to ensure full reproducibility?

Yes

Is the study design appropriate and is the work technically sound?

Yes

Are the conclusions drawn adequately supported by the results?

Yes

Are sufficient details of methods and analysis provided to allow replication by others?

Yes

Reviewer Expertise:

Workforce issues in a broader context.

I confirm that I have read this submission and believe that I have an appropriate level of expertise to confirm that it is of an acceptable scientific standard, however I have significant reservations, as outlined above.

# Reviewer report: Reviewer response for version 1

**DOI:** 10.21956/mep.20953.r34837

**Published:** 2023-10-06

**Authors:** Ayoola Awosika

**Affiliations:** 1 University of Illinois Chicago, Chicago, Illinois, USA

The study done is commendable and highly relevant to the body of knowledge in the scientific community. 

 Most of the evidence stated in the study are coherent and consistent with existing literatures.

 Overall, the study delved into the how anticipatory stress could affect;

1. medical student's -to-residency placement (ideally but the author compared it to post-residency) which in my view it's too far a jump, which could potentially lead to skewed data, due to inability of medical students to give an objective view of what the real-world post-residency job hunting could be like.
2. It compared AS in resident- to- job hunting post-residency.
3. It also compared PD hindsight view having gone through same experience.

Couple of observations:

- The author needs to reword the topic of this article to reflect AS in all concerned (medical students, residents and PD). Extensive details about this comment can be found in the comment box of highlighted portions of the pdf attached.   
- Few factual statements need citation. find comment in pdf attached.
- The idea of hidden curriculum is not clear; it could also vary from persons-to-persons. Consider discussing what it entails for your readers. And how this can potentially create AS for medical students.
- In the discussion section, consider expanding on some of the mitigation strategies like;
  - 1. Incorporating career exploration module sprinkle throughout Undergraduate Medical education and GME. Having a survey that constantly check their preparedness for specialty choice and life after medical school.
  - 2. Encouraging moonlighting early enough during residency training. 
  - 3. The training institution exploring the services of human personnel coach right from day 1 of residency, training resident on how o job hunt, creating an appealing CV, training on how to make early decision (family, location, creating financial plan and goals etc)

Finally, the discussion section or Introduction should consider expatiating on the intercession between physiological and psychological mechanisms for AS.

Have any limitations of the research been acknowledged?

Yes

Is the work clearly and accurately presented and does it cite the current literature?

Yes

If applicable, is the statistical analysis and its interpretation appropriate?

Partly

Are all the source data underlying the results available to ensure full reproducibility?

Yes

Is the study design appropriate and is the work technically sound?

Partly

Are the conclusions drawn adequately supported by the results?

Yes

Are sufficient details of methods and analysis provided to allow replication by others?

Yes

Reviewer Expertise:

Medical education, Cardiometabolic diseases, Pharmacology, Clinical trials

I confirm that I have read this submission and believe that I have an appropriate level of expertise to confirm that it is of an acceptable scientific standard.

# Reviewer report: Reviewer response for version 1

**DOI:** 10.21956/mep.20953.r33719

**Published:** 2023-10-05

**Authors:** Aisha Rafi

**Affiliations:** 1 Shifa Tameer e Millat University, Islamabad, Pakistan

The study aims to explore the construct of anticipatory stress on finding a job after residency. The study addressed the problem of anticipatory stress from medical students, residents, and former program directors.

 There is a consistency between the title, purpose, and research question. The anticipatory stress regarding job prospects after residency and its impact on learner well-being was explored via semi-structured interviews based on the perceived stress scale and primary appraisal/secondary appraisal scale.

 he results of the study showed that the perspectives unique to medical students were proximal issues overburdened with the concern of employment. Similarly, competing residency programs and yearly hiring were the  perspectives of residents and project directors. The study concluded that AS has physical and psychological manifestations on performance anxiety, and pursuing additional training.  

 Although the study has been correctly designed and is technically sound and data is robust to generate sufficient results, but a certain discrepancy has been seen in the instrument used for collection of data mentioned in abstract and the method section of the manuscript. The semi-structured interview is mentioned in abstract and cognitive interview technique is mentioned in methods. The cognitive interview technique should be detailed so that other researchers should be able to conduct the research and reproduce the results in the future study. The semi-structured interview is mentioned in abstract and cognitive interview technique is mentioned in main body text. The cognitive interview technique used in the study needs elaboration to help other researchers to conduct the research in future

Have any limitations of the research been acknowledged?

Yes

Is the work clearly and accurately presented and does it cite the current literature?

Yes

If applicable, is the statistical analysis and its interpretation appropriate?

Not applicable

Are all the source data underlying the results available to ensure full reproducibility?

Yes

Is the study design appropriate and is the work technically sound?

Partly

Are the conclusions drawn adequately supported by the results?

Yes

Are sufficient details of methods and analysis provided to allow replication by others?

No

Reviewer Expertise:

Medical Education, Anatomy

I confirm that I have read this submission and believe that I have an appropriate level of expertise to confirm that it is of an acceptable scientific standard, however I have significant reservations, as outlined above.
